# Nanocellulose-Assisted Construction of Multifunctional MXene-Based Aerogels with Engineering Biomimetic Texture for Pressure Sensor and Compressible Electrode

**DOI:** 10.1007/s40820-023-01073-x

**Published:** 2023-04-10

**Authors:** Ting Xu, Qun Song, Kun Liu, Huayu Liu, Junjie Pan, Wei Liu, Lin Dai, Meng Zhang, Yaxuan Wang, Chuanling Si, Haishun Du, Kai Zhang

**Affiliations:** 1 State Key Laboratory of Biobased Fiber Manufacturing Technology, Tianjin Key Laboratory of Pulp and Paper, Tianjin University of Science and Technology, Tianjin, 300457 People’s Republic of China; 2 Sustainable Materials and Chemistry, Department of Wood Technology and Wood-Based Composites, University of Göttingen, 37077 Göttingen, Germany; 3 Department of Chemical Engineering, Auburn University, Auburn, AL 36849 USA; 4 State Key Laboratory of Bio-Based Materials and Green Papermaking, Qilu University of Technology (Shandong Academy of Sciences), 3501 Daxue Road, Jinan, 250353 People’s Republic of China

**Keywords:** Nanocellulose, Aerogels, MXene, Supercapacitors, Pressure sensors

## Abstract

Multifunctional architecture with intriguing structural design is highly desired for realizing the promising performances in wearable sensors and flexible energy storage devices. Cellulose nanofiber (CNF) is employed for assisting in building conductive, hyperelastic, and ultralight Ti_3_C_2_T_x_ MXene hybrid aerogels with oriented tracheid-like texture. The biomimetic hybrid aerogels are constructed by a facile bidirectional freezing strategy with CNF, carbon nanotube (CNT), and MXene based on synergistic electrostatic interaction and hydrogen bonding. Entangled CNF and CNT “mortars” bonded with MXene “bricks” of the tracheid structure produce good interfacial binding, and superior mechanical strength (up to 80% compressibility and extraordinary fatigue resistance of 1000 cycles at 50% strain). Benefiting from the biomimetic texture, CNF/CNT/MXene aerogel shows ultralow density of 7.48 mg cm^−3^ and excellent electrical conductivity (~ 2400 S m^−1^). Used as pressure sensors, such aerogels exhibit appealing sensitivity performance with the linear sensitivity up to 817.3 kPa^−1^, which affords their application in monitoring body surface information and detecting human motion. Furthermore, the aerogels can also act as electrode materials of compressive solid-state supercapacitors that reveal satisfactory electrochemical performance (849.2 mF cm^−2^ at 0.8 mA cm^−2^) and superior long cycle compression performance (88% after 10,000 cycles at a compressive strain of 30%).

**Supplementary Information:**

The online version contains supplementary material available at 10.1007/s40820-023-01073-x.

## Highlights

- Hyperelastic and superlight multifunctional MXene/nanocellulose composite aerogels with high conductivity are designed by constructing biomimetic texture.
- The MXene/nanocellulose aerogels as flexible pressure sensors exhibit appealing linear sensitivity performance (817.3 kPa^−1^).
- The as-prepared compressible supercapacitor with MXene/nanocellulose electrodes reveals superior electrochemical performance (849.2 mF cm^−2^ at 0.8 mA cm^−2^).

**Supplementary Information:**

The online version contains supplementary material available at 10.1007/s40820-023-01073-x.

## Graphical abstract

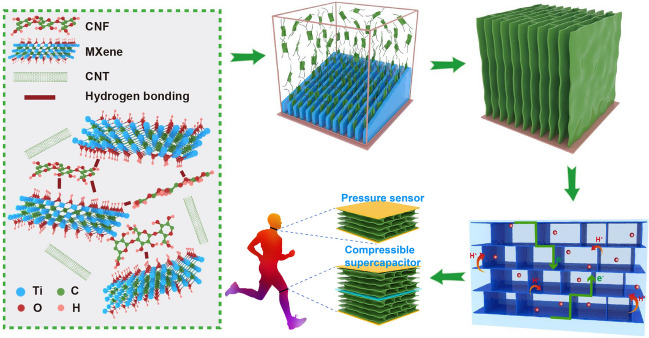

## Introduction

With the booming development of human–computer interaction, the Internet of Things, and wearable electronics, multifunctional materials with superb electrical conductivity and good mechanical properties are emergently desired for flexible sensors and energy storage devices [1–5]. Lightweight and elastic aerogels have been one of the most important candidates for developing high-performance multifunctional platforms due to their tunable structure, low density, and high porosity [6, 7]. The conductive carbon aerogels synthesized from nanocarbons [such as graphene oxide, carbon nanotube (CNT)] or carbonized polymer materials have been demonstrated good performances in the application of constructing flexible sensors and energy storage devices [8–11]. Although carbon aerogels show good conductivity, their components need to be further reduced or carbonized, which is prone to severe volume shrinkage, resulting in poor mechanical properties.

Transition-metal carbon/nitride (MXene)-based aerogels are appealing for flexible electronics because of their highly porous structure and large internal surface areas [12–15]. MXene aerogels can be derived by direct freeze-drying or supercritical drying of MXene hydrogels. However, the relatively weak interactions between MXenes sheets derived from the surface terminations (–O, –OH, and –F groups) cannot effectively balance the electrostatic repulsive interactions and the strong interplanar van der Waals interactions between MXene nanosheets, which make delaminated MXene nanosheets inevitably begin to aggregate and restack during the aerogel fabrication processes [16, 17]. The compact self-stacking structure hinders electrons transport and restricts stress transfer in 3D frameworks, bringing about poor conductivity and mechanical properties. Therefore, the introduction of hydrogen bonding, covalent bonding, or van der Waals forces by low dimensional nanomaterials [18–21] or polymers [22, 23] is useful for the construction of high-performance MXene aerogels.

At present, the development of multifunctional platforms by MXene-based aerogels is still in its infancy. Effective structure design has been verified to be of great significance in constructing functional carbon aerogels [10, 24]. Specifically, the cellulose nanofiber (CNF)/lignin-based carbon aerogel with ordered tracheid-like texture was fabricated and revealed high performance in the application of pressure sensors and flexible electrodes due to the effective stress transfer [24]. The tailored internal structure in the 3D scaffold was demonstrated to be very suitable to construct functional materials with excellent mechanical compressibility and fatigue resistance. To this end, designing MXene-based aerogels with engineering tailored architecture and components to facilitate electrons transport and stress transfer should be an effective route to obtain ideal multifunctional framework.

CNF with sustainability, high aspect ratio, and abundant hydroxyl groups as a component of functional materials has been attracted increasing attention [25–28]. Herein, inspired by the hierarchical tracheid structure in nature wood, multifunctional CNF/CNT/MXene aerogels with engineering biomimetic texture are fabricated by facile bidirectional freezing strategy, demonstrating good mechanical strength and superior electrical conductivity. To this aim, three key considerations are proposed: (1) the electrostatic repulsion between CNF and MXene can avoid restacking of MXene nanosheets, (2) the entangled CNF and CNT “mortars” bonded with MXene “bricks” of the tracheid structure produce good interfacial interactions, and (3) the ordered engineering structure could effectively enable electrons transport and stress transfer. The constructed CNF/CNT/MXene aerogels as pressure sensors exhibit appealing sensing performance, which have broad applications in capturing human bio signals. The aerogels can also act as electrode materials for compressive solid-state supercapacitors with satisfactory electrochemical performance and superior long cycle compression performance.

## Experimental Section

### Materials

TEMPO-oxidized CNF suspension was purchased from Woodelfbio Co., Ltd. (China), whose length ranged from 1 to 5 µm and the diameter from 10 to 20 nm. Multi-walled carbon nanotubes were purchased from Beijing HWRK Chemical Co., Ltd. (China). Ti_3_AlC_2_ powder was provided by Jilin 11 technology Co., Ltd. (China). PVA and Lithium fluoride (LiF) were purchased from Aladdin (China). Sulfuric acid (H_2_SO_4_) and hydrochloric acid (HCl) were bought from Beijing Chemical Reagents Co., Ltd. (China).

### Preparation of Ti_3_C_2_T_x_

Ti_3_C_2_T_x_ was synthesized by selectively etching the Ti_3_C_2_T_x_ MAX phase with LiF/HCl solution [29]. Typically, LiF powder (1.6 g) was dissolved in 9 M HCl (20 mL) in a Teflon vessel and stirred for 10 min to ensure the dissolution of LiF. Then, Ti_3_AlC_2_ powder (1 g) was gradually added into the above LiF/HCl etching solution, and the mixture was continuously stirred for 48 h at 35 °C to obtain a stable suspension. The etched Ti_3_C_2_T_x_ suspension was repeatedly washed with deionized water by centrifugation at 3500 rpm for 5 min until the pH of the obtained suspension was adjusted to 6. The suspension was conducted by ultrasonic treatment for 30 min to obtain exfoliated Ti_3_C_2_T_x_. Finally, the Ti_3_C_2_T_x_ dispersion was further centrifuged at 3500 rpm for 1 h to obtain the delaminated Ti_3_C_2_T_x_ sheets.

### Preparation of CNF/CNT/MXene Aerogel

CNF dispersion (4 mg mL^−1^) and CNT suspension (4 mg mL^−1^) were mixed at the CNF/CNT mass ratios of 1:1, 2:1 and 3:1. The mixtures of CNF and CNT were stirred and sonicated for 1 h to form a homogeneous suspension. Then, the above mixtures of three different ratios were respectively added into MXene dispersion (8 mg mL^−1^) at the CNF/CNT/MXene mass ratios of 1:1:8, 2:1:7 and 3:1:6, followed by stirring and ultrasonicating for 1 h. The uniform suspension was poured into a silicone mold placed on a copper bridge, one end of which was inserted into liquid nitrogen, and the other end was immersed into water at room temperature to form a temperature gradient on the copper surface. Subsequently, the sample was freeze dried at − 50 °C under a pressure of 0.2 mbar for 72 h in a freeze dryer to obtain CNF/CNT/MXene aerogel.

### Characterization

The morphology of MXene was characterized by transmission electron microscopy (TEM, Talos G2 200X) and Bruker multimode atomic force microscope (AFM). The microstructure of CNF/CNT/MXene aerogel was observed under a scanning electron microscopy (SEM, JEOL JSM-IT300LV, Japan). X-ray diffraction (XRD) analysis was carried out using a DMAX2500 Riguku diffractometer with Cu K_α_ radiation in the 2θ range of 5°–50° at a scan rate of 5° min^−1^. The surface elemental and chemical bonding in CNF/CNT/MXene aerogel were evaluated by X-ray photoelectron spectroscopy (XPS, Thermo Fisher K-Alpha, USA). The chemical structure was recorded by Fourier Transform Infrared Spectrometer (FTIR, FTIR-650, China). The electrical conductivity is determined with a 4-probeTech RST-8 resistivity meter (China). Compression and cycling tests were carried out using a universal tester (Lishi LD23.53).

Assembly and sensing performance testing of strain sensor: The highly sensitive strain sensor was fabricated by placing the CNF/CNT/MXene aerogel between two pieces of copper foil adhered to a bandage. The electrical current and sensing measurements of aerogel were recorded on the electrochemical workstation.

The sensitivity (S, kPa^−1^) is calculated according to the following Eq. (1):

1 $${\text{S}} =\updelta \left( {\Delta {\mathrm{I}}/{\mathrm{I}}_{0} } \right)/\updelta {\text{P}}$$

where I_0_ is the initial current (A), ΔI is the relative change in current (A), P is the applied pressure (kPa).

Electrochemical measurements: All electrochemical tests were performed on a Chenhua CHI 660E electrochemical workstation. Three-electrode electrochemical measurement was tested in 1.0 M H_2_SO_4_ aqueous solution, by using Ag/AgCl electrode and platinum sheet as reference electrode and counter electrode, respectively. The CNF/CNT/MXene aerogel was directly used as working electrode without additional conductive additive. Cyclic voltammetry (CV) and galvanostatic charge–discharge (GCD) were measured at room temperature. The specific capacitance of the electrode was calculated on the basis of GCD curves according to the following Eq. (2) [30]:

2 $${\text{C}} = {\text{I}}\Delta {\mathrm{t}}/{\mathrm{m}}\Delta {\mathrm{V}}$$

where I is the discharge current (A), Δt is the discharge time (s), m is the mass of electroactive material (g), and ΔV is the voltage range of discharge (V).

The solid-state supercapacitor was fabricated using two pieces of CNF/CNT/MXene aerogel as electrodes, a cellulose paper as separator, PVA/H_2_SO_4_ gel as solid electrolyte, and two pieces of copper foil as current collector. To prepare PVA/H_2_SO_4_ gel, 10 g PVA was mixed with 100 mL deionized water and stirring for 6 h at 95 °C. After cooling, 1 g of concentrated H_2_SO_4_ was added into the above mixture. Subsequently, the CNF/CNT/MXene aerogels were placed onto copper foils and coated with PVA/H_2_SO_4_ gel. These two electrodes were separated by a cellulose paper and assembled into a sandwich architecture supercapacitor.

The areal specific capacitance of electrode (C_S_) was calculated on the basis of GCD curves according to the following Eq. (3):

3 $${\text{C}}_{{\mathrm{s}}} = 2{\mathrm{I}}\Delta {\mathrm{t}}/{\mathrm{S}}\Delta {\text{V}}$$

The capacitance of the supercapacitor (C_device_) was calculated according to the following Eq. (4):

4 $${\mathrm{C}}_{{{\text{device}}}} = {\text{C}}_{{\mathrm{s}}} /2 = {\text{I}}\Delta {\mathrm{t}}/{\mathrm{S}}\Delta {\text{V}}$$

The energy density and power density were respectively calculated according to the following equations:

5 $${\text{E}} = 0.5{\text{C}}_{{{\mathrm{device}}}} \left( {\Delta {\text{V}}} \right)^{2} /3600$$

6 $${\text{P}} = {\text{E}} \times 3600/\Delta {\text{t}}$$

where I is the discharge current (A), Δt is the discharge time (s), S is the area accessible to the electrolyte, and ΔV is the voltage range of discharge (V).

## Results and Discussion

### Preparation of CNF/CNT/MXene Aerogels and Structural Characterizations

**Fig. 1:**
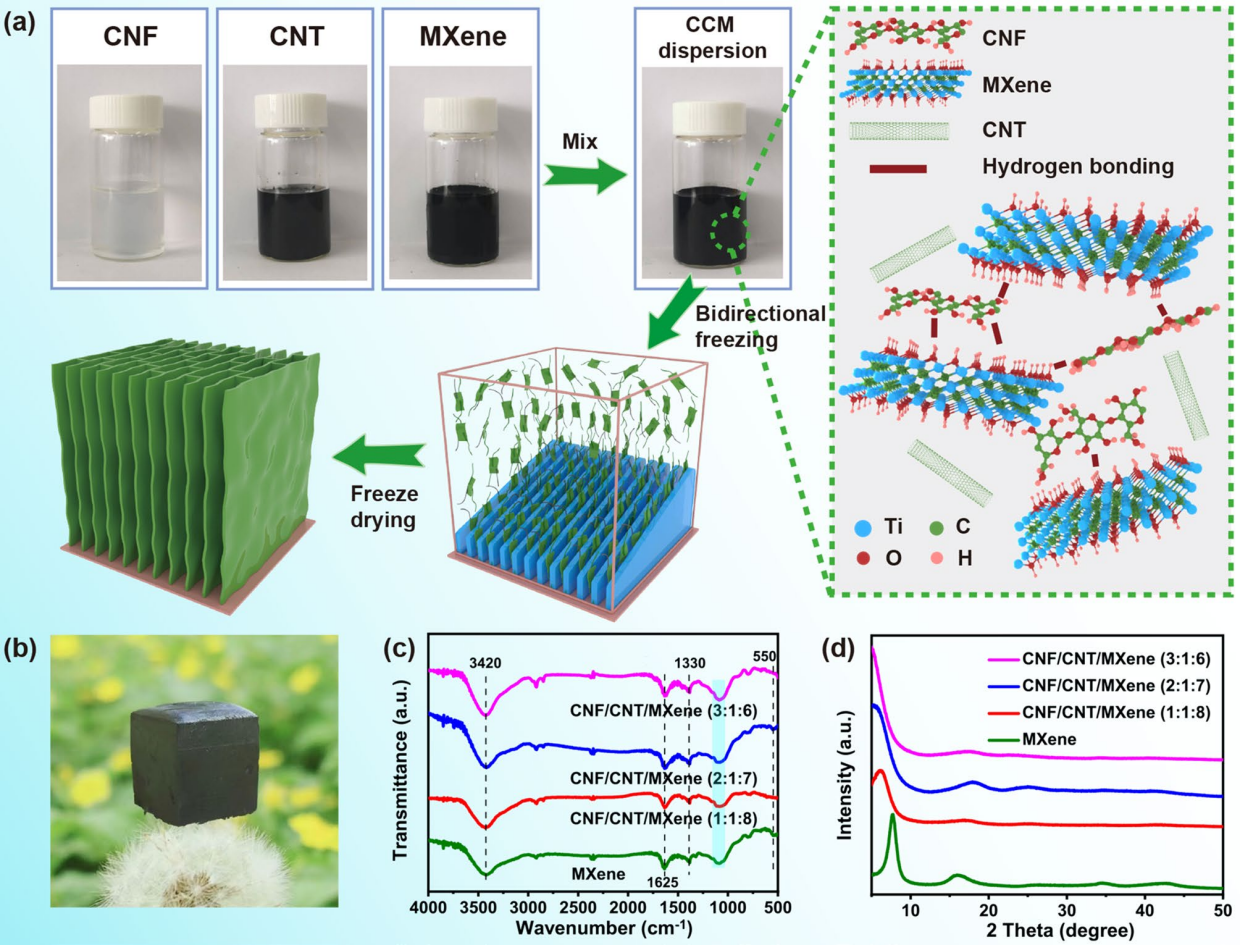
**a** Schematic illustration for the fabrication process of CNF/CNT/MXene aerogels. **b** Photo image of the lightweight CNF/CNT/MXene aerogel on the top of a dandelion. **c** FTIR and **d** XRD patterns of MXene and different CNF/CNT/MXene aerogels

To form ordered porous and robust CNF/CNT/MXene architectures, CNF is employed for tailoring the interaction between surface chemical groups and suppressing restacking of MXene sheets. Moreover, the presence of multi-walled CNT can improve the conductivity of CNF/CNT/MXene aerogels [31]. Figure 1a illustrates the fabrication process of CNF/CNT/MXene aerogels. The high aspect ratio CNF with a diameter of (10–15 nm) (Fig. S1a) was prepared by 2,2,6,6-tetramethylpiperidine-1-oxyl-oxide (TEMPO) oxidation and subsequent high-pressure homogenization. The MXene sheets (Fig. S1b, c) were obtained by etching and exfoliating their Ti_3_AlC_2_ MAX phase precursor with LiF/HCl solution to selectively remove the Al layers. Moreover, the AFM image of Ti_3_C_2_T_x_ nanosheets reveals their average thickness of 1.4 nm and length of 3–4 μm. The typical XRD patterns of raw material (Ti_3_AlC_2_ MAX), etched Ti_3_C_2_T_x_, and Ti_3_C_2_T_x_ are shown in Fig. S2. By comparing the position of the peaks between the Ti_3_AlC_2_ MAX precursor and Ti_3_C_2_T_x_ MXene, it could be found that the (002) peak shifted from 9.7° of MAX to 7.3° of MXene, indicating that the interlayer distance increased, which was attributed to the fact that the Al layer was removed and surface terminations were introduced [14]. The CNT was added into the CNF dispersion to ensure the uniform dispersion of CNT. In the dispersion of CNF/CNT, the electrostatic repulsion formed between the carboxyl groups on the CNF chain prevented the agglomeration of CNT [32]. Then, MXene suspension and the CNF/CNT dispersion were mixed to obtain the CNF/CNT/MXene (CCM) dispersion. In this process, MXene nanosheets with abundant surface oxygen-containing functional groups strongly interacted with CNF through hydrogen bonding [33]. The CNF inserted the interlayer of MXene and prevented the aggregation of MXene nanosheets. Finally, the resultant CCM dispersion suffered from the bidirectional freezing and freeze-dried process to obtain the CNF/CNT/MXene aerogel. As shown in Fig. 1a, by applying the bidirectional temperature gradient to CCM dispersion, water molecules nucleated at the frozen surface and grew along the direction of the temperature gradient. The intertwined CNF, CNT, and MXene were repelled by ice crystals and squeezed onto the interface, then ordered porous aerogel was obtained after freeze-drying. To investigate the effects of CNF component on porous structure, the CNF/CNT/MXene aerogels with different mass ratios and CNT/MXene aerogel were prepared, as shown in Table S1. The as-prepared CNF/CNT/MXene aerogel demonstrates robust architecture and ultralow density, which can rest on the tips of a dandelion (Fig. 1b). The interactions among the components of CNF/CNT/MXene aerogels were investigated in detail. In the FTIR spectrum (Fig. 1c), the typical bands of 550, 1625, and 3440 cm^−1^ in MXene and CNF/CNT/MXene aerogels correspond to Ti–O, C=O, and –OH groups, respectively [34]. For MXene, the peaks at 1040 and 1330 cm^−1^ are attributed to the stretching vibration of C–O and –OH groups, confirming oxygen-containing groups on the surface of MXene. And the band relating to the stretching vibration of –CO– shifts to a lower wavenumber (from 1050 to 1030 cm^−1^), which indicate the strong hydrogen bonding interaction between CNF and MXene nanosheets [35]. The structural evolutions from MXene to the CNF/CNT/MXene aerogels were monitored with XRD patterns (Figs. 1d and S3). In the XRD profile of MXene, a prominent peak is found at 7.3°, corresponding to the (002) peak of Ti_3_C_2_T_x_ MXene, and the peak of 16.0° is referred to the (004) plane of Ti_3_C_2_T_x_ MXene [36]. The characteristic peak (002) in the patterns of CNF/CNT/MXene aerogels shows a left shift, indicating that the interlayer *d*-spacing of MXene nanosheets is enlarged and CNF/CNT successfully insert the interlayers of MXene nanosheets. The XPS spectrum of CNF/CNT/MXene aerogels was also performed to study the chemical bonding states and elemental compositions of the aerogels. As demonstrated in Fig. S4a, the XPS patterns show that the CNF/CNT/MXene aerogels contain the element of Ti, C, O, and F. The C 1*s* spectrum of CNF/CNT/MXene (2:1:7) aerogel is shown in Fig. S4b. A typical peak at 281.8 eV (Ti–C) is observed, the 287.5 eV of C=O, 286.2 eV of C–O, and 284.6 eV of (C–C) are well-maintained in the CNF/CNT/MXene aerogels.

**Fig. 2:**
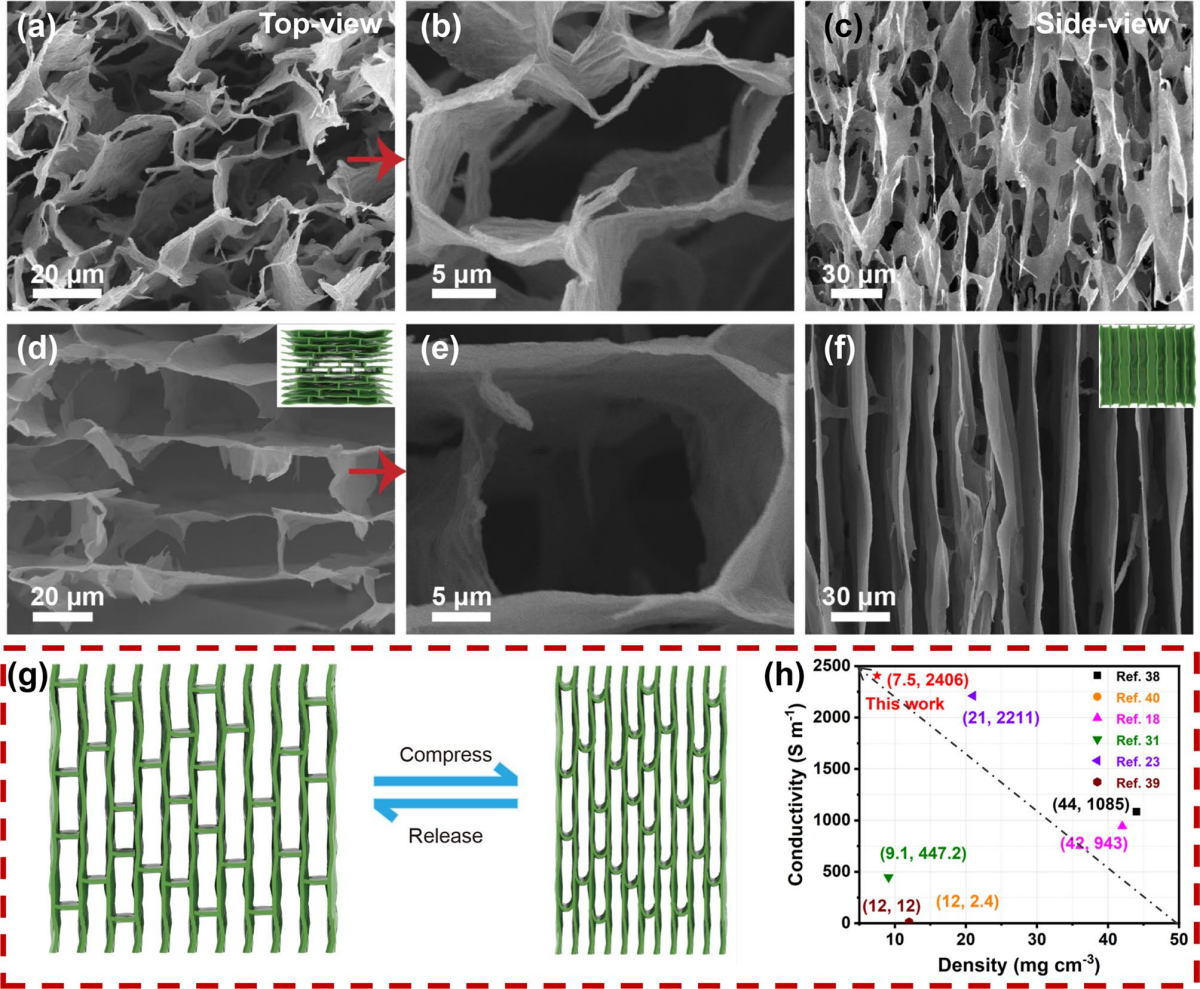
**a**, **b** The top-view and **c** side-view SEM images of CNT/MXene (1:7) aerogel. **d**, **e** The top-view and **f** side-view SEM images of CNF/CNT/MXene (2:1:7) aerogel, the inset is schematic diagram of the pore structure. **g** Schematic illustration of compression and release process for CNF/CNT/MXene aerogel (2:1:7). **h** Comparison of the conductivity with other MXene-based aerogels

Figure 2a–f show the SEM images of CNT/MXene (1:7) and CNF/CNT/MXene (2:1:7) aerogels. The CNT/MXene (1:7) aerogel without CNF component exhibits a loosely disordered porous structure (Fig. 2a, b), and the connections between MXene sheets are not continuous (Fig. 2c), which makes the fragile and brittle architecture collapse easily by large strain compression. Conversely, the CNF/CNT/MXene (2:1:7) aerogel demonstrates the ordered tracheid network (Fig. 2d, e) and smooth cell walls structure (Fig. 2f), exhibiting anisotropic porous structure. These structural differences can be attributed to the intrinsic interactions among the components in aerogels. For CNT/MXene aerogel, the existing relatively weak π–π interaction between CNT and MXene, and the relatively weak intrinsic interaction of MXene sheets make the poor structural continuity [37]. For CNF/CNT/MXene aerogel, numerous hydrogen bonds can be formed between the groups of –COOH/–OH on CNF and –OH on the surface of MXene. The CNF acts as a coupling agent to enhance the assembled MXene sheets. Therefore, the CNF entangled with CNT interconnects adjacent MXene nanosheets to form the continuous and ordered network. Furthermore, the CNF/CNT/MXene (1:1:8) and CNF/CNT/MXene (3:1:6) aerogels also display similar pore structures, as shown in Fig. S5. However, the continuity of the biomimetic structure is broken when the CNF content is too high (Fig. S5c, d), which mainly because of the excessive CNF would join the main body of the MXene “bricks”. The designed elaborately pore structure endows CNF/CNT/MXene (2:1:7) aerogel with superior mechanical compressibility and resilience, as shown in Fig. S6. And Fig. 2g exhibits the schematic illustration of stress transfer in internal pore structure during the compression-recovery process. Benefiting from the ordered tracheid network, the CNF/CNT/MXene (2:1:7) aerogel with the low density of 7.5 mg cm^−3^ also exhibits superhigh conductivity of 2400 S m^−1^, which is superior to that of Ti_3_C_2_T_x_ MXene/reduced graphene oxide hybrid aerogel [38], Ti_3_C_2_T_x_/CNT hybrid aerogel [18], CNF/ammonium polyphosphate/Ti_3_C_2_T_x_ composite aerogels [39], and etc. [29, 40], as shown in Fig. 2h. The conductivities of the aerogel along vertical and longitudinal directions were also measured to be 2080 and 2300 S m^−1^, respectively. The similar conductivities of three directions could be ascribed to the exceptional continuity and interconnection of the tracheid network.

### Mechanical Properties of CNF/CNT/MXene Aerogels

**Fig. 3:**
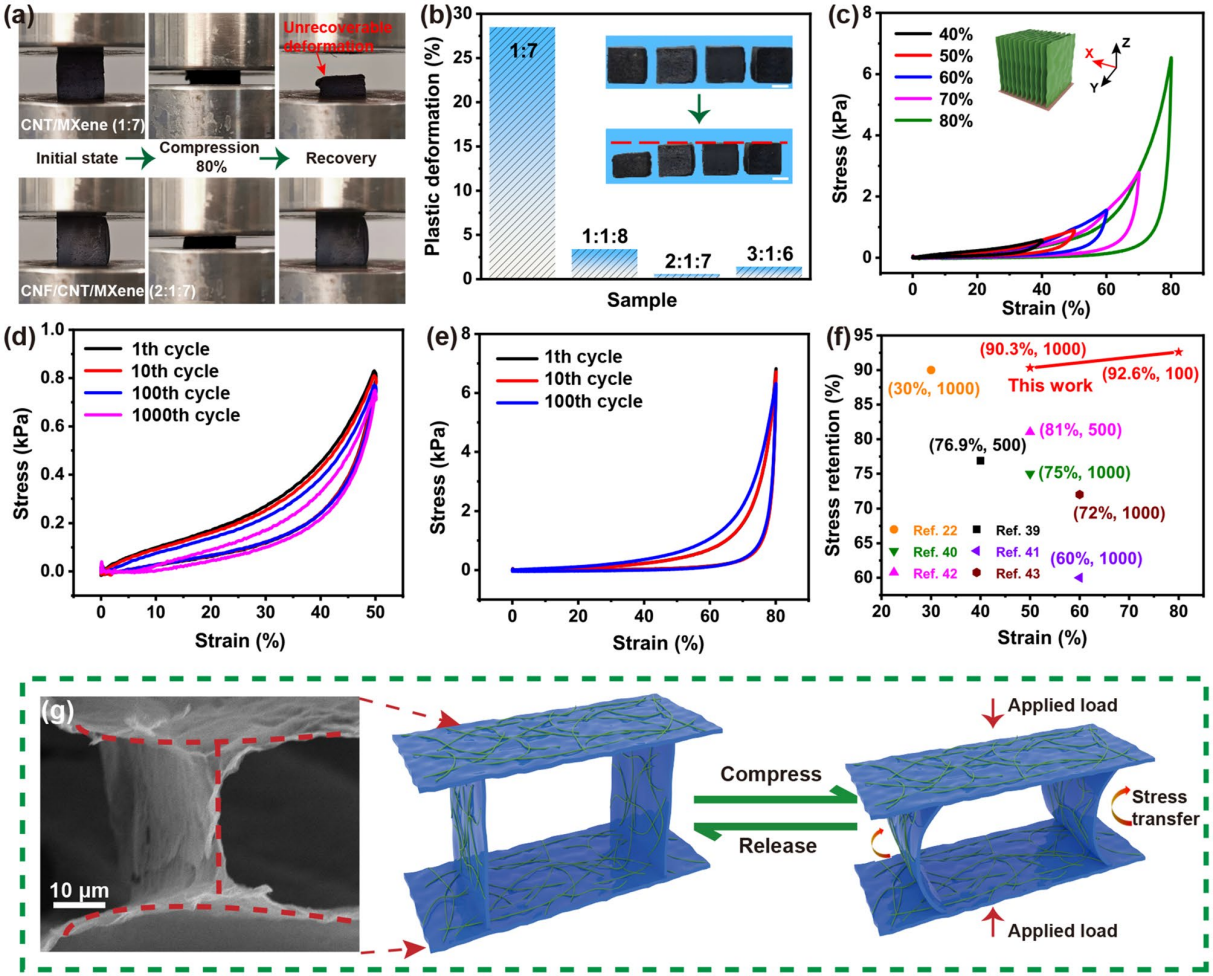
**a** Experimental photographs for the first compression cycle of CNT/MXene (1:7) and CNF/CNT/MXene (2:1:7) aerogels. **b** Histograms of irreversible deformation percentages after the first cycle. The inset shows the height contrast photos of samples after the first cycle (the scale bar is 1 cm). **c** Stress–strain curves of CNF/CNT/MXene (2:1:7) aerogel at 40%–80% compression strains in X-direction (the inset shows the direction of compression). Stress–strain curves **d** at 50% strain for 1000 cycles and **e** at 80% strain for 100 cycles. **f** Comparison of the stress retention of CNF/CNT/MXene (2:1:7) aerogel with those of MXene- and carbon-based aerogels. **g** Mechanism illustration of the compressive deformation of the CNF/CNT/MXene (2:1:7) aerogel

The compressibility and fatigue resistance are investigated to explore the effects of CNF component and the designed biomimetic porous structure on the mechanical strength of CNF/CNT/MXene aerogel. The compressibility and fatigue resistance of CNT/MXene (Fig. S7) and CNF/CNT/MXene aerogels (Figs. 3 and S8) are demonstrated. As shown in Fig. 3a and b, The CNT/MXene (1:7) aerogel exhibit severe plastic deformation (irreversible deformation of up to 28.5%) at 80% compression strain. In contrast, the CNF/CNT/MXene aerogels can undergo broad compression strain and show much smaller unrecoverable plastic deformation of merely 0.6% (Fig. 3b) owing to the addition of CNF and ordered tracheid structure. Figure 3c demonstrates the stress–strain curves at 40–80% compression strain of CNF/CNT/MXene (2:1:7) aerogels in X-direction. With increasing compression strains the profiles become gradually steepened. Particularly, the recovery curves almost overlap under the low strain (ε < 60%). This is because the distance between the aerogel sheets decreases with increasing strains in the initial elastic region but the microstructure of CNF/CNT/MXene (2:1:7) aerogel remains stable. Moreover, the fatigue resistance of CNF/CNT/MXene (2:1:7) aerogel is evaluated by cyclic compressions at a strain of 50% and 80%. Remarkably, the CNF/CNT/MXene (2:1:7) aerogel can withstand long-term compression for 1000 cycles, showing high stress retention of 90.3% at the strain of 50% (Fig. 3d). Even at 80% compression strain, the aerogel can keep a stress retention of 92.6% after 100 cycles of compression (Fig. 3e), which further proves the excellent compressibility and elasticity of CNF/CNT/MXene aerogel. Therefore, the tracheid structure including the entangled CNF and CNT “mortars” bonded with MXene “bricks” endows the aerogels with high compressibility and elasticity. As shown in Fig. 3f, the mechanical performance of CNF/CNT/MXene aerogel is superior to MXene/aramid nanofibers composite aerogel [20], many carbon-based compressible aerogels [41–45].

To illustrate the superior structural stability of CNF/CNT/MXene aerogels, the elastic and compressible mechanism is proposed in Fig. 3g. In the structural model, the regular mortars-bricks structure of CNF/CNT/MXene aerogel makes it possible to avoid slipping and splitting along the perpendicular direction of compression, which is more conducive to the storage of elastic energy [24]. Moreover, the entangled CNF and CNT a role in reinforcing the MXene-based architecture by interconnecting the MXene nanosheets. During the compression process, the anisotropic pore structure is deformable, facilitating the stress transformation and compression to large strains. The continuous and dense CNF/CNT/MXene hybrid architecture like a bow makes the aerogels elastic. The stress–strain curves of CNF/CNT/MXene (2:1:7) aerogel from Y- and Z-directions were examined, as shown in Fig. S9. As can be seen, the structure of the aerogels was destroyed at 25% (Z-direction) and 32% (Y-direction), respectively. It could be attributed to the directional porous structure of anisotropic aerogels [40].

### Pressure and Strain Sensing Performances of CNF/CNT/MXene Aerogels

**Fig. 4:**
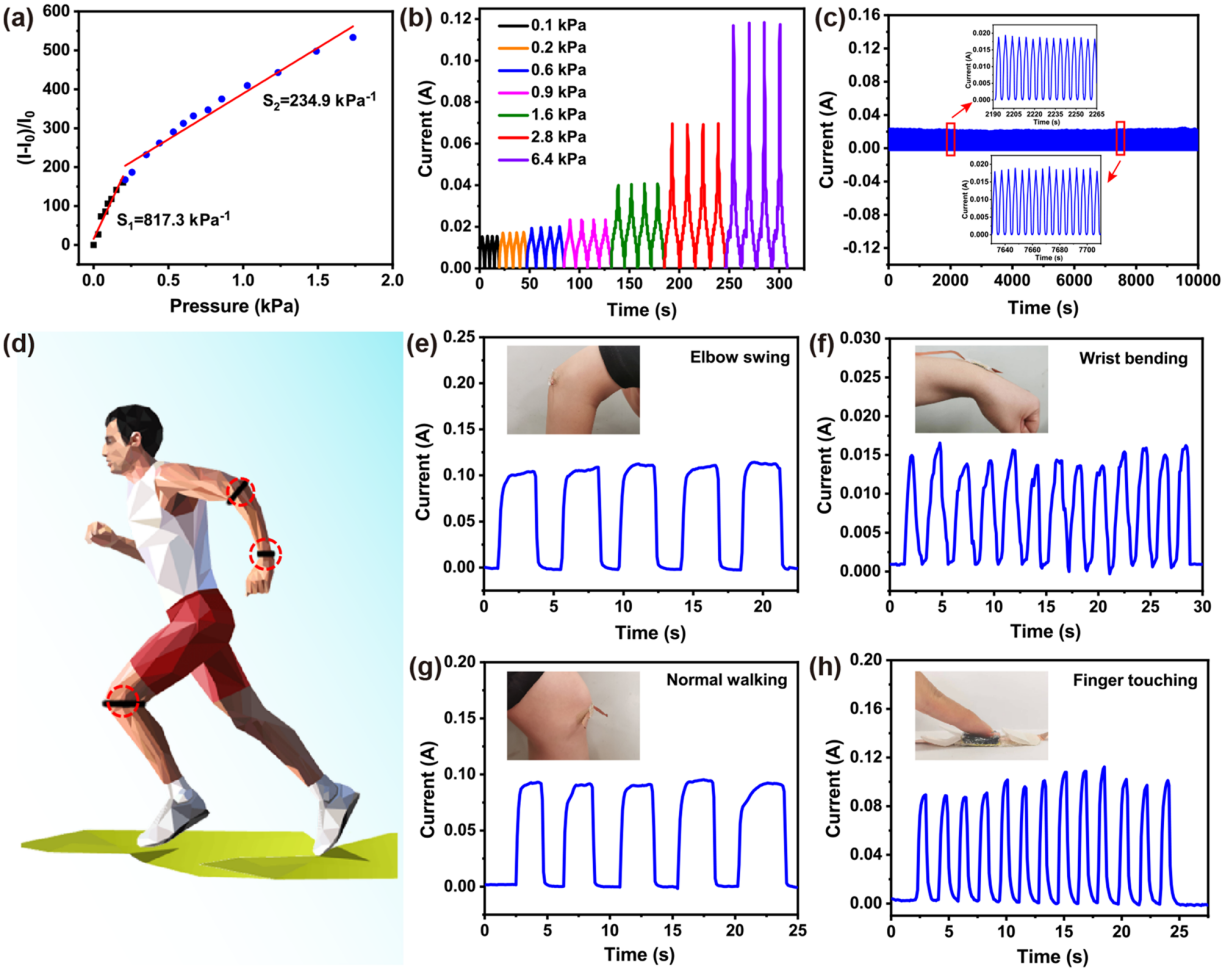
**a** The relationship between the change of the relative current and the linear sensitivity of the pressure sensor. **b** Current response at various pressures of 0.1–6.4 kPa. **c** Current stability at 20% strain for 2000 cycles. **d** Illustration diagram of application in human behavior monitoring. Current signals from **e** elbow swing, **f** wrist bending, **g** normal working, and **h** finger touching

Excellent conductivity, mechanical robustness, large compressive strain, and superior fatigue resistance make the CNF/CNT/MXene (2:1:7) aerogel a promising candidate for the flexible pressure sensor. During the process of compression, the bulb gradually turned bright in the closed circuit (Fig. S10). It is turn out that the distance between the aerogels gradually decreased, resulting in enhanced electric current and lower electrical resistance. To explore its piezoelectric properties, a sensor is fabricated with a sandwich structure by CNF/CNT/MXene (2:1:7) aerogel between two pieces of polyethylene terephthalate substrates [10], and the corresponding sensor is shown in Fig. S11. Figure S12 demonstrates the real-time current response to different pressures (0–10,000 Pa). The current intensity continuously rises with increasing pressure, demonstrating its potential application in detecting pressure. The sensing sensitivity (S) is a significant performance parameter of flexible pressure sensor, which characterizes the sensitivity of the sensor to external stress. As S = (ΔI/I_0_)/ΔP [46, 47], where I_0_ is the current without the external pressure, ΔI is the relative change of the current, and ΔP is the change external pressure. As demonstrated in Fig. 4a, like most reported sensors, the current change versus pressure curve of the CNF/CNT/MXene (2:1:7) aerogel piezosensor can be divided into two linear regions [48]. In the region of 0–200 Pa, the sensitivity of S_1_ is 817.3 kPa^−1^. And in the region of 200–1,500 Pa, S_2_ is up to 234.9 kPa^−1^. The sensitivities are superior to CNF/CNT/reduced graphene oxide (RGO) carbon aerogels (5.61 kPa^−1^) [10], melamine sponge-MWCNTs@CB (48.26 kPa^−1^) [49], wood-derived CNFs/lignin carbon aerogels (5.16 kPa^−1^) [24], etc. The higher sensitivity of the CNF/CNT/MXene (2:1:7) aerogel-based pressure sensor can be attributed to the following reasons: (1) The entangled nano-CNFs and CNTs on the surface of the CNF/CNT/MXene aerogel enhance the roughness, and the larger contact area increases the number of conductive paths under the action of external force. (2) The unique tracheid structure of the aerogel makes the inner pore size and the distance between the pores uniformly decrease under the action of external force, then the closely contacted nanowalls in CNF/CNT/MXene aerogel can form lots of conductive paths. Figure 4b shows the real-time current responses of the CNF/CNT/MXene (2:1:7) aerogel for 5 cycles at compression strains from 20% to 80%. As expected, the current increased significantly during compression and decreased rapidly during release, indicating a fast current responsive capability with compressive strain for the CNF/CNT/MXene aerogel. Moreover, the 2000 cycles test experiments at 30% strain on the CNF/CNT/MXene aerogel-based sensor were performed, as shown in Fig. 4c. It was found that the CNF/CNT/MXene aerogel-based sensor exhibited good stability, and the initial current intensity was basically maintained. Furthermore, the sensor reveals rapid response (74 ms) and recovery (50 ms) abilities, as shown in Fig. S13. Generally, the sensing performance of CNF/CNT/MXene aerogels with those compressible MXene-based aerogels and carbon aerogels was compared (Table S3), exhibiting superior sensitivity, response/recovery time, and long-term stability.

Based on the superior mechanical sensing properties of CNF/CNT/MXene aerogel, it was applied to monitor body movement and physiological state (Fig. 4d). First, the sensor was attached to the elbow, wrist, and knee to monitor joint flexion movement. As exhibited in Fig. 4e, the current gradually enhances as the angle of the elbow swing increases. At a fixed swing amplitude, the current remains relatively constant. And when the swing is repeated at the same swing angle, the current curve shows good repeatability. Similarly, for the bending of the wrist, normal working, and finger touching (Fig. 4f–h), the current response values also show the same trend and demonstrate good cycling stability. In addition, when attaching the sensor to the human throat, it can detect the current change when speaking a word such as “MXene” or “Chemical” (Fig. S14).

### Electrochemical Performance of CNF/CNT/MXene Aerogels

The ordered porous structure, superior fatigue resistance, and good electrical conductivity of the CNF/CNT/MXene aerogels make them potential as electrodes for compressible supercapacitors [50]. Firstly, the electrochemical performance of CNF/CNT/MXene aerogels was evaluated in a three-electrode system in 1 M H_2_SO_4_ electrolyte (Figs. S15–S17). As shown in Fig. S15, CNF/CNT/MXene (2:1:7) aerogel electrode showed the largest specific capacitance of 215.8 F g^−1^ at 0.3 A g^−1^. This is attributed to the excellent conductivity, continuous pore wall structure, and good hydrophilic of CNF/CNT/MXene (2:1:7) aerogel that facilitate the electrons and ions transport. When a sufficiently small amount of MXene nanosheets is added to the CNF and CNT systems, MXene nanosheets are evenly dispersed in the 3D network structure resulting in a large rectangular curve [51]. In comparison, excessive MXene at higher contents will destroy the 3D pore structure of aerogels, resulting in the decrease of carbon aerogel capacitance. Figure S15a, b exhibits cyclic voltammetry (CV) curves of the CNF/CNT/MXene (2:1:7) aerogel electrode at various scan rates from 2 to 500 mV s^−1^. Clearly, all the CV curves present a rectangular-like shape, indicating good electrochemical reversibility [52]. Otherwise, obvious redox peaks were observed at 2–50 mV s^−1^, which corresponds to the pseudo capacitance behavior of MXene. Figure S15c demonstrates the galvanostatic charge/discharge (GCD) curves of CNF/CNT/MXene (2:1:7) aerogel electrode at current density of 0.3–1.0 A g^−1^. Even at 1.0 A g^−1^, the specific capacitance of the electrode remains 146.9 F g^−1^. The good electrochemical reversibility and rate performance make the CNF/CNT/MXene (2:1:7) aerogel promising for high-performance supercapacitors.

**Fig. 5:**
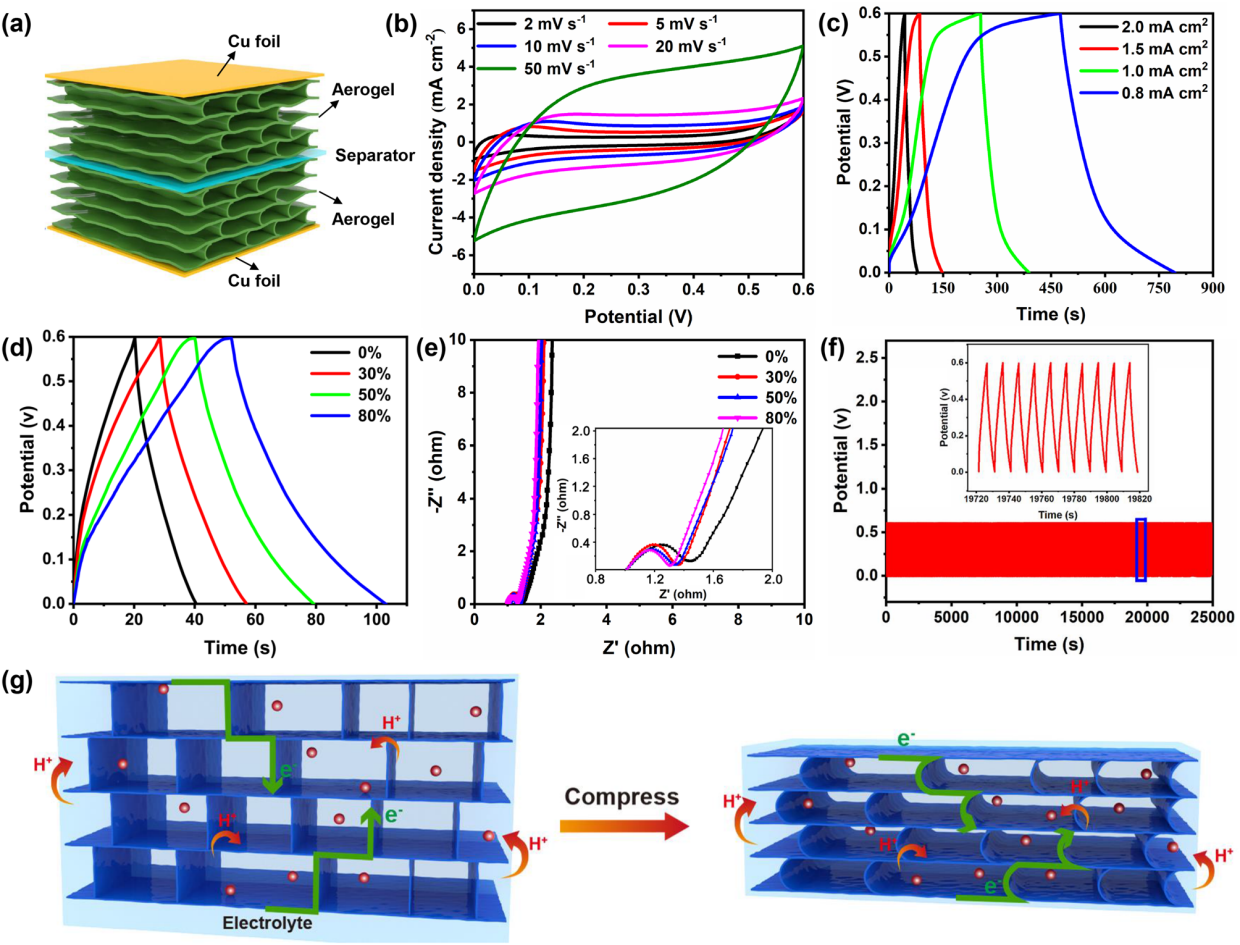
**a** Schematic illustration of the assembled compressible supercapacitor. **b** CV curves of compressible supercapacitor at scan rates of 2–50 mV s^−1^. **c** GCD curves at different areal current densities. **d** GCD curves and **e** Nyquist plots of compressible supercapacitor under various strains from 0 to 80%. **f** Cycling stability of solid-state compressible supercapacitors over 10,000 cycles under 30% strain. **g** The process illustration of ions and electrons transport in electrodes before and after compression

To show the potential application of CNF/CNT/MXene aerogel as the compressible electrode, the sandwich-like compressible supercapacitors (Fig. 5a) were assembled with the same two CNF/CNT/MXene (2:1:7) aerogel electrodes and polyvinyl alcohol/H_2_SO_4_ (PVA/H_2_SO_4_) gel electrolyte. By tuning the thickness of the compressible supercapacitors, the strains of CNF/CNT/MXene aerogel can be facilely controlled. Figure 5b shows the CV profiles of the compressible supercapacitors under different strains. The CV curves of obtained solid supercapacitors showed similar shapes at different scan rates (2–50 mV s^−1^), indicating good rate-adaptive performance and electrochemical reversibility. The solid-state supercapacitors at different current densities showed good capacitive behaviors based on their almost symmetrical triangle shapes within GCD curves (Fig. 5c). Based on these GCD curves, the electrodes delivered an areal specific capacitance of 849.2 mF cm^−2^ at a current density of 0.8 mA cm^−2^, which is higher than that of CNF/CNT/RGO carbon aerogel (109.4 mF cm^−2^ at 0.4 mA cm^−2^) [10], MXene-RGO composite aerogel (34.6 mF cm^−2^ at 1 mV s^−1^) [20], and comparable to the 3D printed carbon aerogel (870.3 mF cm^−2^) [53], and other aerogels [54, 55]. As shown in Fig. S18, the specific capacitance retention of the solid-state supercapacitors is as high as 88% even after 10,000 charging and discharging cycles at a current density of 10 mA cm^−2^, highlighting its excellent cycle stability. Furthermore, the assembled solid-state supercapacitors delivered an energy density of about 21.2 μWh cm^−2^ at a power density of 240.0 μW cm^−2^. The excellent electrochemical performance should be attributed to the highly porous structure of the CNF/CNT/MXene aerogel, which enable the enhanced interface and high contact surface area between the microcells inside the electrode and the electrolyte, thereby reducing the interface transfer resistance and improving the capacitance [56]. Owing to the high mechanical compressibility of CNF/CNT/MXene aerogel, assembled solid-state supercapacitors are expected to be highly compressible. To evaluate the compressibility, solid supercapacitors were tested under different compressive strains. It is obvious that the devices can withstand up to 80% strain without structural damage. The GCD curves expanded as the strain increased from 0 to 80%, and the capacitive performance of the device was significantly enhanced (Fig. 5d). Similarly, the area of CV curves became larger with increasing compression strains (Fig. S19). The capacitance retention under different strains is shown in Fig. S20. To understand the mechanism of the performance difference, the EIS of solid-state supercapacitor under various strains were performed and the Nyquist plots are presented in Fig. 5e. It is seen that all the Nyquist plots display similar shape consisting of an arc in the higher frequency region followed by a spike at low frequency. The charge transfer resistance (R_ct_) is found to decrease with the increase of the strains, showing gradually enhanced charge transfer capability at the electrode/electrolyte interfaces due to the improved conductivity at higher strains [57, 58]. Therefore, increasing compression should improve the interface contact between electrolyte and electrode, thereby increasing the accessible electrochemical position and accelerating ion transfer (Fig. 5g). Apart from the outstanding energy density and power density, the solid-state symmetric supercapacitor also exhibited excellent cycling stability under compressive strain. At a strain of 30%, almost of its initial capacitance was retained after 10,000 consecutive cycles, suggesting the excellent cycling stability of assembled devices under high compression (Fig. 5f). The strategy of using compressive CNF/CNT/MXene aerogel as composite electrodes provides a novel and feasible method for the preparation of compressible supercapacitors with high electrochemical and mechanical properties.

## Conclusions

In summary, multifunctional conductive nanocellulose/carbon nanotube/MXene aerogels have been designed and fabricated with ultralight and superior mechanical strength by facile bidirectional freezing. Supporting CNF and CNT in the composite aerogels effectively inhibited the stacking of MXene nanosheets, resulting in the formation of regularly arranged tracheid-like architecture. The abundant oriented pore structure not only effectively transferred stress, but also contributed to the transportation of electrons and ions. Being used as electrodes for strain sensors, the composite aerogel exhibited good linear sensitivity of 817.3 kPa^−1^, demonstrating application prospects in monitoring body movement and physiology. Moreover, the CNF/CNT/MXene aerogel can be used for solid-state compressible supercapacitors, and displayed significant electrochemical performances, including high capacitance (849.2 mF cm^−2^ at 0.8 mA cm^−2^), outstanding cycling stability (88% capacitance retention after 10,000 cycles), and superior mechanical flexibility. It is believed that this research can provide a facile but effective method for constructing high-performance and promising multifunctional platforms.

### Supplementary Information

Below is the link to the electronic supplementary material.

Supplementary file1 (PDF 1562 KB)
