# Supplementary material for: Nanocellulose-Assisted Construction of Multifunctional MXene-Based Aerogels with Engineering Biomimetic Texture for Pressure Sensor and Compressible Electrode
Source: Nanomicro Lett. 2023 Apr 10;15:98. doi: 10.1007/s40820-023-01073-x (PMC10086089; doi:10.1007/s40820-023-01073-x)
Supplement: Supplementary file 1 — Supplementary file1 (PDF 1562 KB) [file 40820_2023_1073_MOESM1_ESM.pdf]

Supporting Information for

Nanocellulose-Assisted Construction of Multifunctional MXene-Based Aerogels with Engineering Biomimetic Texture for Pressure Sensor and Compressible Electrode

Ting Xu[1, †], Qun Song[2, †], Kun Liu[1, †], Huayu Liu[1, †], Junjie Pan[2], Wei Liu[1, 2], Lin Dai[1], Meng Zhang[1], Yaxuan Wang[1], Chuanling Si[1, 4] *, Haishun Du[3,]*, and Kai Zhang[2,]*

[1]State Key Laboratory of Biobased Fiber Manufacturing Technology, Tianjin Key Laboratory of Pulp and Paper, Tianjin University of Science and Technology, Tianjin 300457, P. R. China

[2]Sustainable Materials and Chemistry, Department of Wood Technology and Wood-based Composites, University of Göttingen, Göttingen D-37077, Germany

[3]Department of Chemical Engineering, Auburn University, Auburn AL-36849, USA

[4]State Key Laboratory of Bio-based Materials and Green Papermaking, Qilu University of Technology (Shandong Academy of Sciences), 3501 Daxue Road, Jinan 250353, P. R. China

[†]Ting Xu, Qun Song, Kun Liu, and Huayu Liu contributed equally to this work.

*Corresponding authors. E-mail: kai.zhang@uni-goettingen.de (K. Zhang); sichli@tust.edu.cn (C. Si); hzd0024@auburn.edu (H. Du)

Supplementary Figures

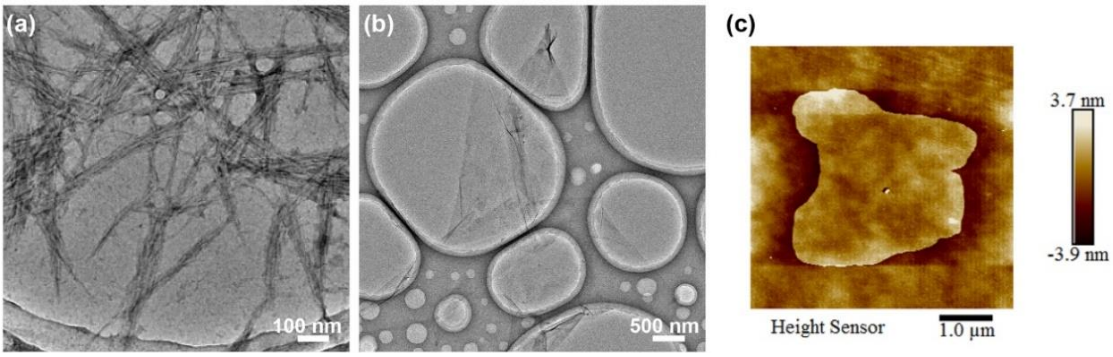

Fig. S1 The TEM images of (**a**) CNF and (**b**) MXene, and (**c**) AFM image of MXene

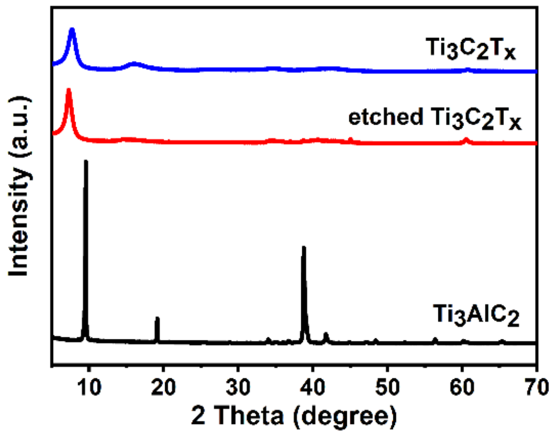

Fig. S2 XRD patterns of raw material (Ti_3AlC_2), etched $Ti_3C_2T_x$, and $Ti_3C_2T_x$

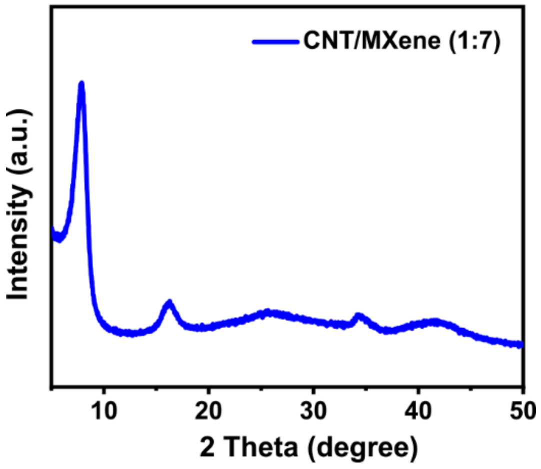

Fig. S3 XRD pattern of CNT/MXene (1:7) aerogel

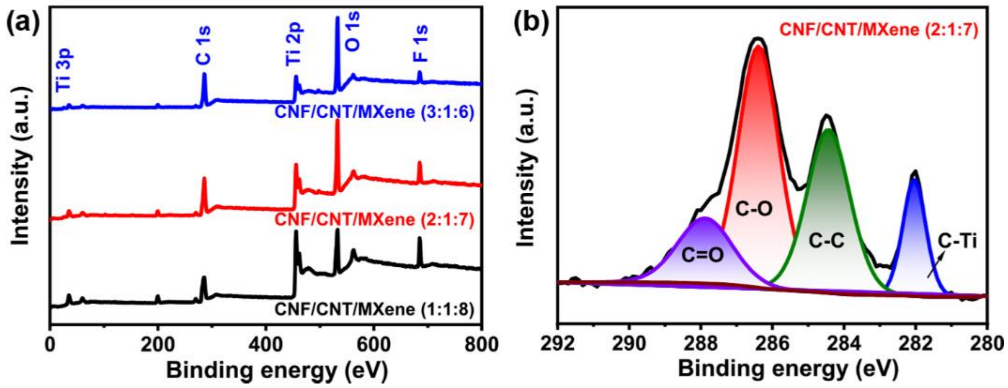

Fig. S4 XPS spectrum of (**a**) CNF/CNT/MXene (3:1:6), CNF/CNT/MXene (2:1:7), and CNF/CNT/MXene (1:1:8) aerogels. (**b**) XPS high-resolution C 1s spectra of CNF/CNT/MXene (2:1:7) aerogel

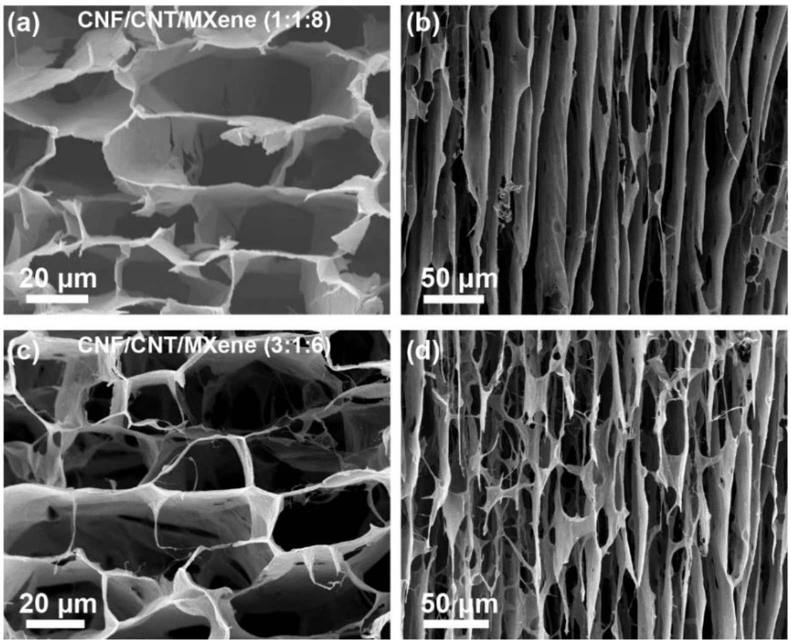

Fig. S5 (**a**) The top-view and (**b**) side-view SEM images of CNF/CNT/MXene (1:1:8) aerogel. (**c**) The top-view and (**d**) side-view SEM images of CNF/CNT/MXene (3:1:6) aerogel

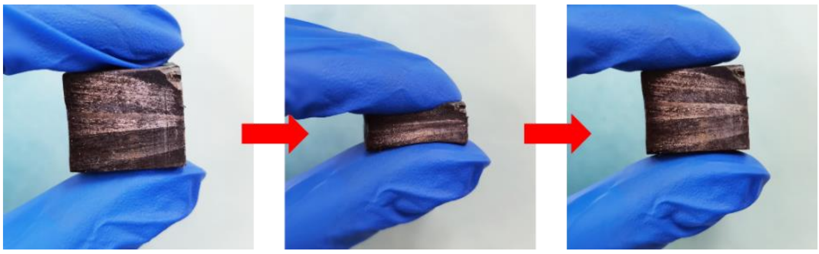

Fig. S6 The optical image of compression and recovery process of the CNF/CNT/MXene (2:1:7) aerogel

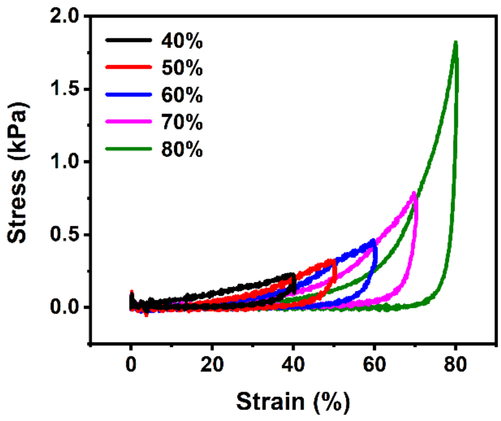

Fig. S7 Stress-strain curves of CNT/MXene (1:7) aerogel at different compression strains

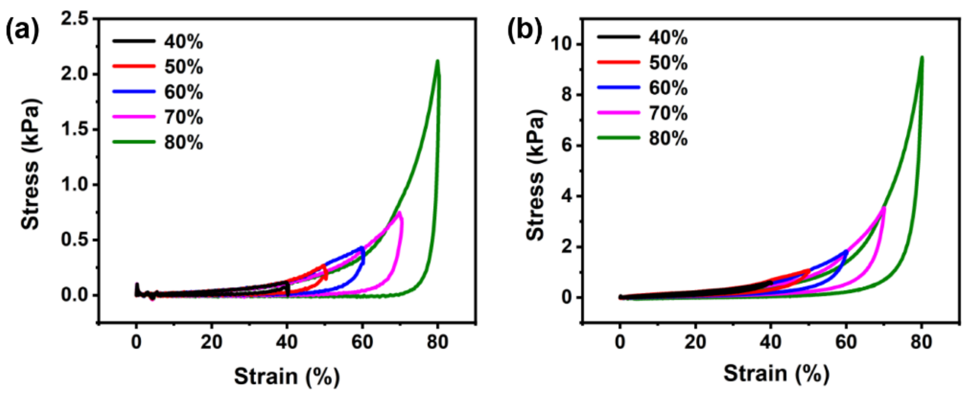

Fig. S8 Stress-strain curves of (**a**) CNF/CNT/MXene (1:1:8) aerogel and (**b**) CNF/CNT/MXene (3:1:6) aerogel at different compression strains

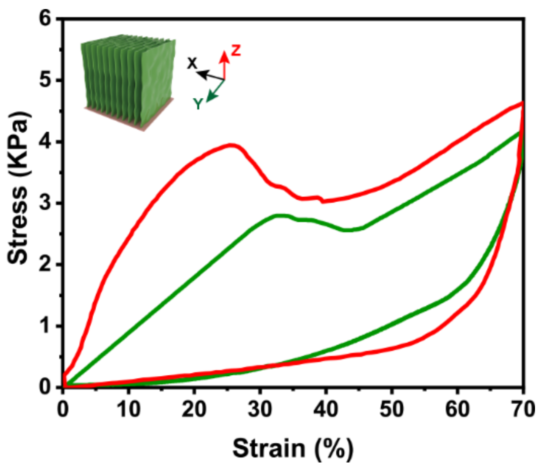

Fig. S9 Stress-strain curves of CNF/CNT/MXene (2:1:7) aerogel in Y-direction and Z-direction

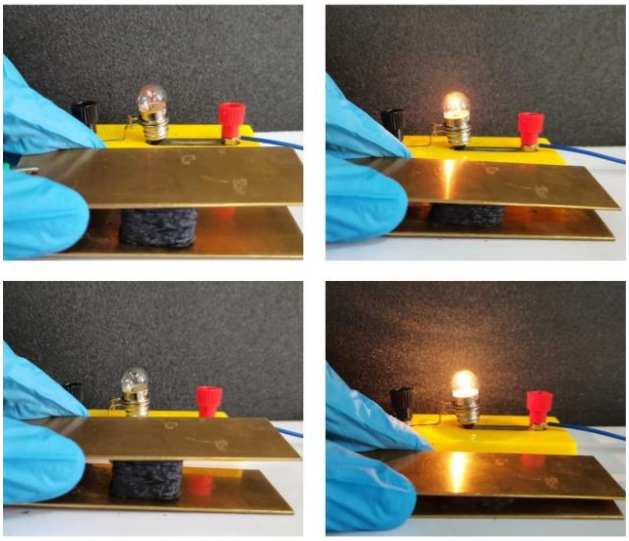

Fig. S10 The changes of bulb brightness under different strains in a closed circuit

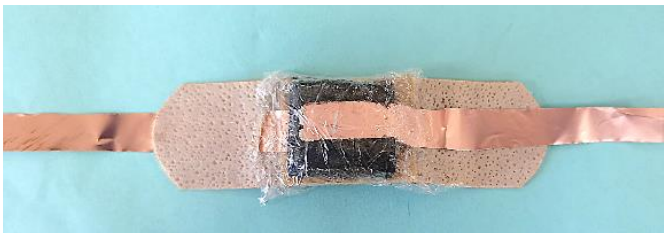

Fig. S11 Digital photo of the assembled sensor

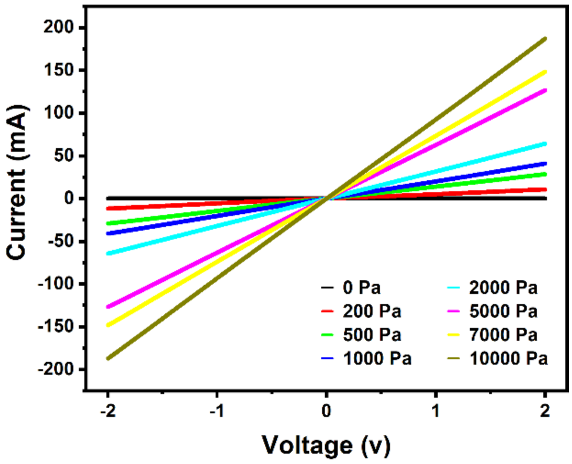

Fig. S12 Current response at different pressures with a voltage ranging from -2 to 2 V

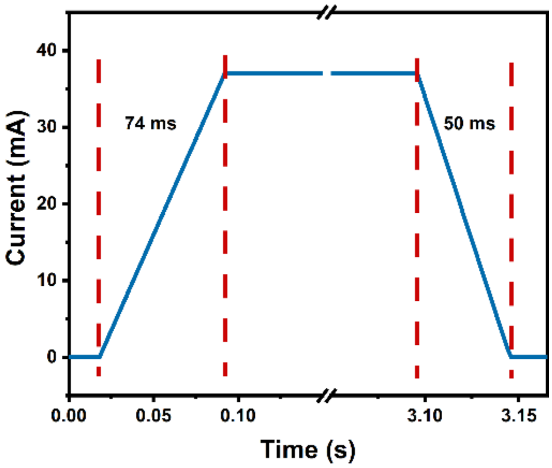

Fig. S13 Response and recovery times of the sensor

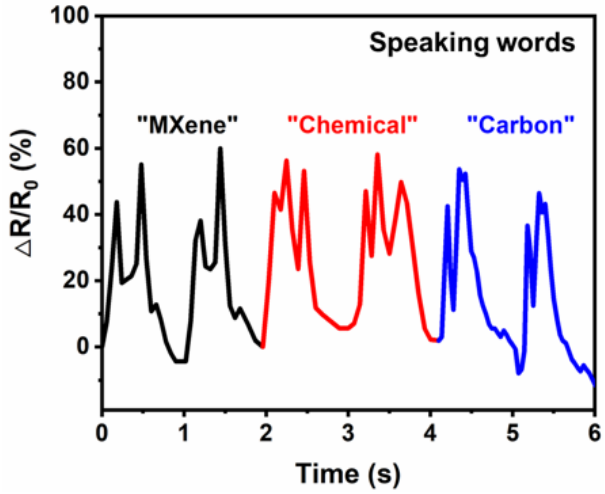

Fig. S14 Current signals from speaking different words

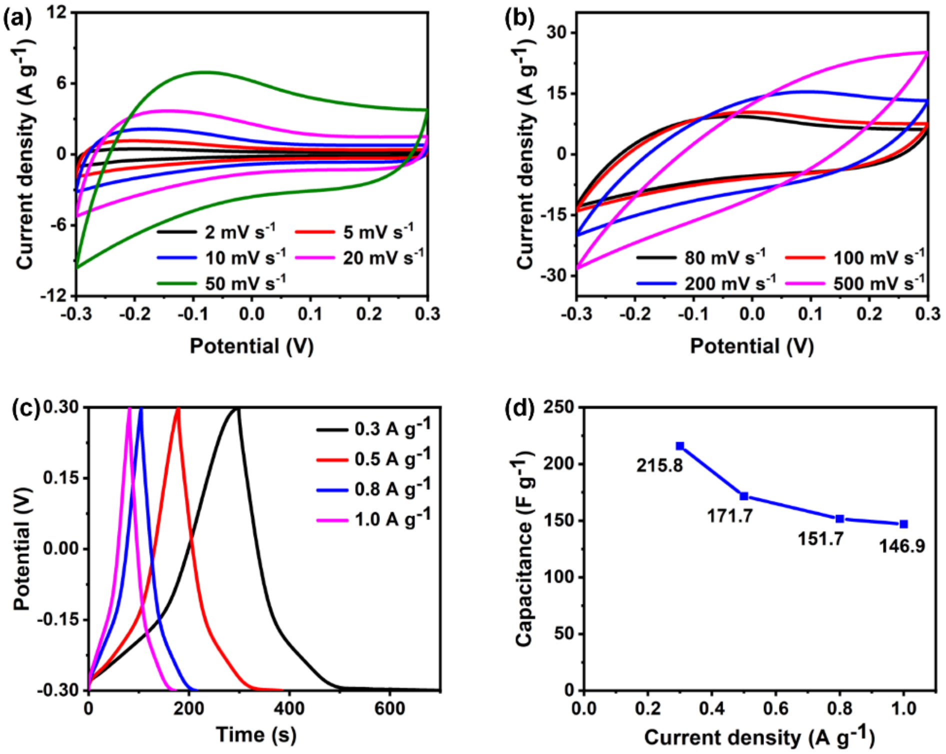

Fig. S15 (**a, b**) CV curves at different scan rates and (**c**) GCD profiles at different current densities of CNF/CNT/MXene (2:1:7) aerogel electrode. (**d**) Specific capacitance of CNF/CNT/MXene (2:1:7) aerogel electrode based on the GCD profiles

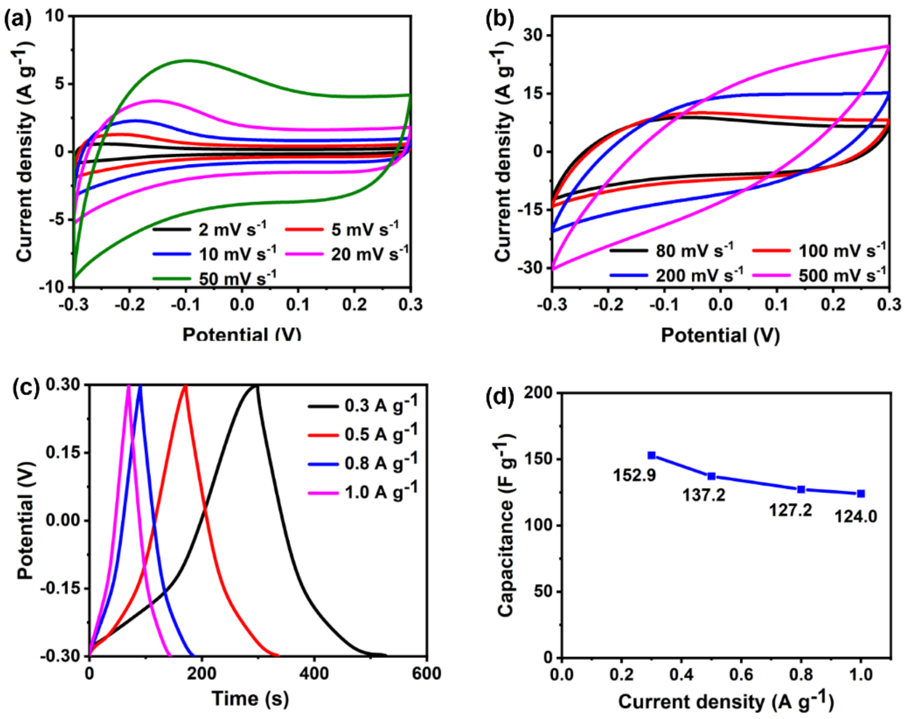

Fig. S16 (**a, b**) CV curves at different scan rates and (**c**) GCD profiles at different current densities of CNF/CNT/MXene (1:1:8) aerogel electrode. (**d**) Specific capacitance of CNF/CNT/MXene (1:1:8) aerogel electrode based on the GCD profile

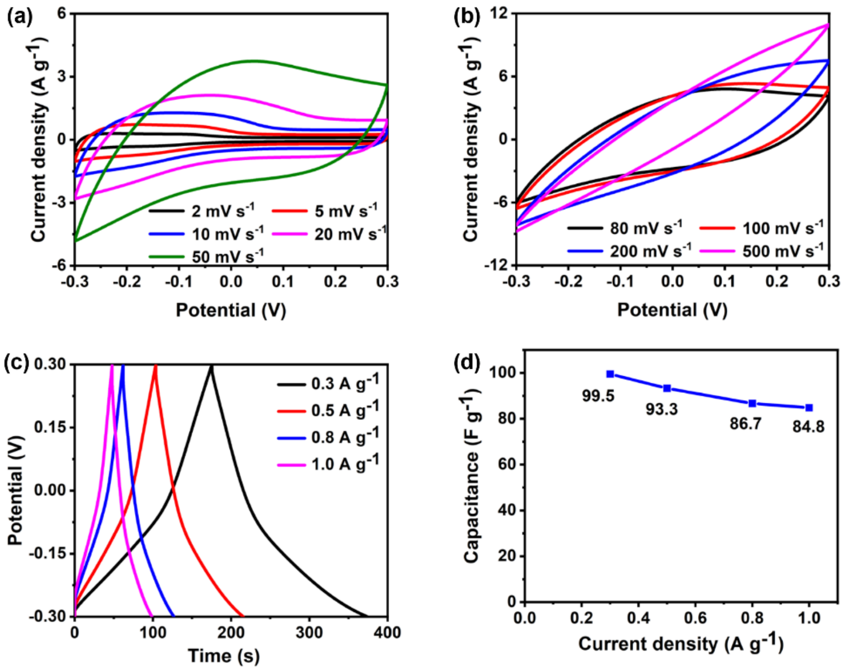

Fig. S17 (**a, b**) CV curves at different scan rates and (**c**) GCD profiles at different current densities of CNF/CNT/MXene (3:1:6) aerogel electrode. (**d**) Specific capacitance of CNF/CNT/MXene (3:1:6) aerogel electrode based on the GCD profiles

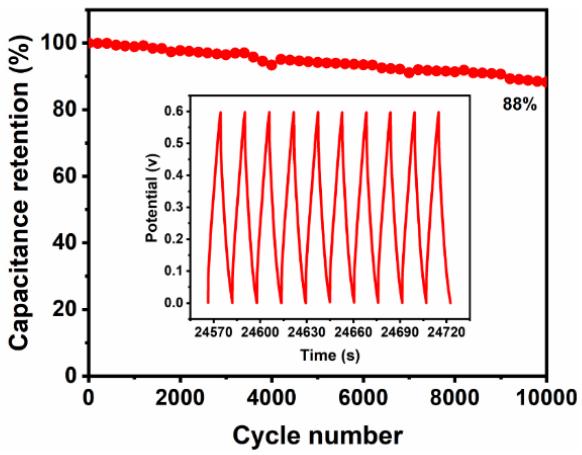

Fig. S18 Cycling stability of compressible supercapacitors over 10000 cycles at 10 mA cm^{-2}

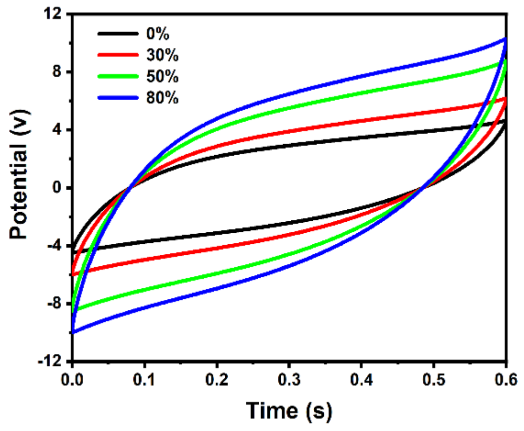

Fig. S19 CV curves of compressible supercapacitor under various strains from 0% to 80%

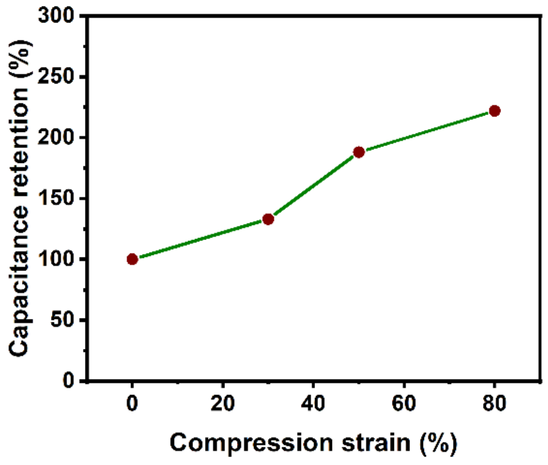

Fig. S20 The capacitance retention under various strains from 0% to 80%

Table S1 The prepared CNF/CNT/MXene aerogels with different mass ratio

CNF/CNT/MXene aerogels	CNF	CNT	MXene
CNF/CNT/MXene (3:1:6)	3	1	6
CNF/CNT/MXene (2:1:7)	2	1	7
CNF/CNT/MXene (1:1:8)	1	1	8
CNT/MXene (1:7)	0	1	7

Table S2 The density and conductivity of different CNF/CNT/MXene aerogels

Sample of aerogels	Density (g cm^{-3})	Conductivity (S m^{-1})
CNF/CNT/MXene (1:1:8)	8.0	1650
CNF/CNT/MXene (2:1:7)	7.5	2400
CNF/CNT/MXene (3:1:6)	7.8	820

Table S3 Comparison of sensor performance of CNF/CNT/MXene aerogel with those compressible MXene-based aerogels and carbon aerogels

Materials	Sensitivity (kPa^{-1})	Pressure range (kPa)	Response/ recovery time (ms)	Long-term stability	Refs.
MXene/silver nanowires aerogel	645.69	0-1	60/144	2000	S1
CNFs/Lignin carbon aerogels	5.16	0-16.89	65/52	1000	S2
Aramid Nanofibers/ MXene Aerogel	128	0-5	320/98	-	S3
MXene/CNF foam	419.7	0-8.04	123/139	10000	S4
MXene/Polyaniline/ Bacterial cellulose aerogel	327.22	0-3	-	-	S5
CNF/CNT/RGO carbon aerogels	5. 61	0-0.21	-	2000	S6
CNF/CNT/MXene aerogel	817.3/234.9	0-0.2/0.2-1.5	74/50	2000	**Our work**

Supplementary References

[S1] L. Bi, Z. Yang, L. Chen, Z. Wu, C. Ye, Compressible AgNWs/$Ti_3C_2T_X$ MXene aerogel-based highly sensitive piezoresistive pressure sensor as versatile electronic skins. J. Mater. Chem. A **8**, 20030-20036 (2020). https://doi.org/10.1039/D0TA07044K

[S2] Z. Chen, H. Zhuo, Y. Hu, H. Lai, L. Liu et al., Wood-derived lightweight and elastic carbon aerogel for pressure sensing and energy storage. Adv. Funct. Mater. **30**, 1910292 (2020). https://doi.org/10.1002/adfm.201910292

[S3] L. Wang, M. Zhang, B. Yang, J. Tan, X. Ding, Highly compressible, thermally stable, light-weight, and robust aramid nanofibers/Ti_3AlC_2 MXene composite aerogel for sensitive pressure sensor. ACS Nano **14**, 10633-10647 (2020). https://doi.org/10.1021/acsnano.0c04888

[S4] T. Su , N. Liu, Y. Gao , D. Lei , L. Wang et al., MXene/cellulose nanofiber-foam based high performance degradable piezoresistive sensor with greatly expanded interlayer distances. Nano Energy **87**, 106151 (2021). https://doi.org/10.1016/j.nanoen.2021.106151

[S5] H. Zhi, X. Zhang, F. Wang, P. Wan, L. Feng, Flexible $Ti_3C_2T_x$ MXene/PANI/Bacterial cellulose aerogel for e-skins and gas sensing. ACS Appl. Mater. Interfaces **13**, 45987-45994 (2021). https://doi.org/10.1021/acsami.1c12991

[S6] H. Liu, T. Xu, C. Cai, K. Liu, W. Liu et al., Multifunctional superelastic, superhydrophilic, and ultralight nanocellulose-based composite carbon aerogels for compressive supercapacitor and strain sensor. Adv. Funct. Mater. **32**, 2113082 (2022). https://doi.org/10.1002/adfm.202113082
